# Evolving Surgical Approaches in the Management of Pancreatic Masses: From Open Resection to Minimally Invasive and Robotic Techniques

**DOI:** 10.7759/cureus.88607

**Published:** 2025-07-23

**Authors:** Cara Mohammed, Patricio Xavier Duran S, Hugh Kolomar, Tanmay Thirth, Simcha Bulmash, Sharvari Vikram Joshi, Tannia Payan Serrano, Greeshma Reddy, Turimula Arpan, Deepika Palegar Thuli, Areehah Zafar Masood, Manju Rai

**Affiliations:** 1 Orthopaedic Surgery, Sangre Grande Hospital, Sangre Grande, TTO; 2 Internal Medicine, Universidad de Cuenca, Cuenca, ECU; 3 Internal Medicine, Niš Clinical Centre, Niš, SRB; 4 Surgery, St. Philomena’s Hospital, Bengaluru, IND; 5 Surgery, Technion-Israel Institute of Technology, The Ruth and Bruce Rappaport Faculty of Medicine, Haifa, ISR; 6 Surgery, Goa Medical College and Hospital, Bambolim, IND; 7 Surgery, Hospital General de Occidente, Universidad de Guadalajara, Jalisco, MEX; 8 Surgery, Sakra World Hospital, Bengaluru, IND; 9 Medicine, Sree Venkateswaraa Medical College and Hospital, Mahabubnagar, IND; 10 Surgery, Osmania Medical College, Hyderabad, IND; 11 Surgery, Ziauddin Medical College, Karachi, PAK; 12 Biotechnology, Shri Venkateshwara University, Amroha, IND

**Keywords:** biliary bypass, endoscopic ultrasound, minimally invasive surgery, multidisciplinary approach, neoadjuvant therapy, pancreatic ductal adenocarcinoma, pancreatic masses, pancreatic resection, postoperative complications, robotic-assisted surgery

## Abstract

Pancreatic masses represent a heterogeneous spectrum of neoplasms, ranging from benign lesions such as serous cystadenomas to malignant tumors such as pancreatic ductal adenocarcinoma (PDAC). Timely and accurate differentiation among these entities is essential for devising effective therapeutic strategies. Recent advancements in diagnostic modalities, surgical techniques, and perioperative care have significantly influenced the management of pancreatic masses. This narrative review synthesizes current literature and clinical practices related to the surgical management of pancreatic masses. It examines the evolution of diagnostic tools, operative approaches, perioperative management, and the integration of emerging technologies in clinical decision-making. Imaging modalities such as contrast-enhanced CT, MRI, and endoscopic ultrasound have improved the precision of diagnosis and staging. Histopathological confirmation remains the gold standard for definitive diagnosis. Surgical options, including partial and total pancreatectomy, are tailored according to tumor type and stage, with resection being the only curative treatment for PDAC. Minimally invasive approaches, such as laparoscopic and robotic-assisted surgeries, offer the benefits of reduced morbidity and faster recovery while maintaining oncological outcomes. Neoadjuvant therapy shows promise in enhancing surgical success and survival in resectable and borderline resectable tumors. Postoperative complications such as pancreatic fistulas, delayed gastric emptying, and endocrine/exocrine insufficiencies are common challenges. For non-resectable tumors, palliative options such as biliary bypass and endoscopic stenting focus on symptom relief and quality of life. The surgical management of pancreatic masses has evolved significantly, driven by advancements in diagnostics, minimally invasive techniques, and supportive perioperative care. Emerging tools such as intraoperative imaging, artificial intelligence, and precision medicine are enabling more personalized treatment plans. A multidisciplinary approach remains critical in optimizing outcomes for patients with pancreatic neoplasms.

## Introduction and background

Pancreatic masses are a diverse group of neoplasms that can significantly impact patient outcomes based on their nature, i.e., benign or malignant. Pancreatic masses represent a clinically significant concern due to their rising global incidence, with pancreatic cancer now ranking among the top causes of cancer-related deaths worldwide. While benign lesions such as serous cystadenomas are relatively rare and often incidental, malignant neoplasms, particularly pancreatic ductal adenocarcinoma (PDAC), constitute the majority and are associated with a five-year survival rate of less than 10%, highlighting their profound clinical impact [1]. The pathophysiology of pancreatic masses varies based on their underlying histology. Benign lesions typically arise from ductal or acinar cell hyperplasia, while malignant tumors such as PDAC develop through a stepwise progression of genetic mutations, most notably involving *KRAS*, *TP53*, *CDKN2A*, and *SMAD4*, that drive dysregulated cell growth, invasion, and stromal desmoplasia [2]. Benign pancreatic tumors, such as serous cystadenomas and intraductal papillary mucinous neoplasms (IPMNs), typically exhibit slow growth and a lower risk of progression to cancer [1]. In contrast, malignant pancreatic masses, including PDAC, are highly aggressive and often present at an advanced stage due to their asymptomatic nature in early development [2]. The prognosis of malignant pancreatic tumors remains poor, with PDAC accounting for over 90% of pancreatic cancer cases and a five-year survival rate below 10% [3]. Distinguishing between benign and malignant pancreatic lesions is crucial for determining the appropriate clinical approach and improving patient outcomes.

Identifying predictors of survival is challenging, as most patients are diagnosed at advanced stages, with only 15-20% of tumors being resectable [3]. Early diagnosis remains difficult, underscoring the need for new biomarkers to detect disease early, monitor recurrence, and assess progression risk [3]. Following surgical resection, surveillance typically includes contrast-enhanced CT or MRI every three to six months during the first two years, and annually thereafter to monitor for recurrence. Serum carbohydrate antigen 19.9 (CA 19-9) levels are also tracked at similar intervals, with rising trends prompting further investigation despite potential confounders such as biliary obstruction or inflammation.

CA 19-9 is the most validated biomarker, with sensitivity and specificity values of 68% and 95%, respectively, a year before diagnosis, and 60% and 90% within six months [2]. Elevated CA 19-9 levels correlate with poorer prognosis, but the low specificity and false-positive rates of CA 19-9 due to conditions such as cholangitis, cirrhosis, or pancreatitis limit its utility. Complementary biomarkers, such as carcinoembryonic antigen (CEA) and cancer antigen 125 (CA 125), improve diagnostic accuracy. Elevated CEA levels are associated with lymph node metastasis, greater tumor burden, and worse prognosis. Novel biomarkers, including the fifth metabolite panel (e.g., acetylspermidine, diacetylspermine) and *KRAS*-mutant circulating tumor DNA, enhance early-stage diagnosis and progression monitoring for advanced stages [4].

Tumor location significantly impacts survival. Tumors in the pancreas head are detected earlier due to obstructive jaundice, while body or tail tumors often present late with advanced-stage disease [5]. Tumor grade is a critical prognostic indicator, with low-grade tumors showing a median survival of 34 months, compared to 15-21 months for high-grade tumors. Key prognostic factors include tumor size (>2 cm), lymph node metastasis, male sex, and older age. Lymphatic metastasis drastically reduces survival, with non-metastatic patients having significantly better outcomes [5].

Timely diagnosis and surgical management are paramount in the effective treatment of pancreatic masses. Early detection of malignant tumors can allow for surgical resection, which remains the only potential curative treatment for PDAC and some other malignant neoplasms [4]. For benign masses, surgical management may be indicated when symptomatic or when there is a high risk of malignant transformation [5]. Diagnostic modalities such as high-resolution imaging and endoscopic ultrasound-guided fine-needle aspiration (EUS-FNA) have advanced the precision in evaluating pancreatic masses, aiding in the differentiation between benign and malignant lesions and guiding surgical planning [6].

This narrative review aims to provide an updated examination of the surgical management of pancreatic masses, including current surgical techniques, perioperative considerations, and potential complications. This review also aims to highlight the challenges and innovations in the surgical field, offering a comprehensive overview for clinicians managing patients with pancreatic lesions. By synthesizing the latest literature, this review seeks to inform best practices and support decision-making processes in the management of both benign and malignant pancreatic tumors.

## Review

Epidemiology of pancreatic masses

Pancreatic masses encompass a diverse group of neoplasms with varying epidemiological patterns, risk factors, and clinical behaviors. The global incidence and prevalence of pancreatic cancer, particularly PDAC, have steadily increased over the past few decades, with PDAC now representing one of the leading causes of cancer-related mortality worldwide. The incidence rates for pancreatic cancer are estimated to be around 8-10 cases per 100,000 population annually, with higher rates observed in Western countries than in Asia or Africa [7]. According to the Global Cancer Observatory, pancreatic cancer ranks as the 12th most common cancer globally but is the seventh leading cause of cancer death due to its high lethality and late-stage presentation [8]. For neuroendocrine tumors (NETs) of the pancreas, however, the incidence is comparatively lower, accounting for approximately 1-2% of all pancreatic tumors [9].

Risk Factors

The etiology of pancreatic masses involves multiple risk factors, with genetic predisposition, environmental exposures, and lifestyle choices playing critical roles. Family history is a significant predictor, with hereditary syndromes such as *BRCA* mutations, Lynch syndrome, and familial atypical multiple mole melanoma syndrome increasing the likelihood of developing pancreatic neoplasms [10]. In addition to genetic factors, lifestyle risks such as smoking, excessive alcohol consumption, obesity, and chronic pancreatitis are well-established contributors. Smoking, in particular, has been associated with a twofold increased risk of PDAC, with former smokers also at an elevated risk compared to nonsmokers [11]. Dietary factors, such as high fat intake and low consumption of fruits and vegetables, have also been implicated in increasing the susceptibility to pancreatic cancer, although evidence remains mixed [12].

Environmental and occupational exposures to certain chemicals, such as pesticides and heavy metals, have been identified as additional risk factors for pancreatic malignancies [13]. Furthermore, recent studies have highlighted the role of metabolic conditions, including diabetes mellitus, in the pathogenesis of pancreatic cancer, with longstanding diabetes associated with a significantly increased risk of developing PDAC [14]. Addressing these modifiable risk factors could potentially contribute to reducing the incidence of pancreatic masses.

Types of Pancreatic Masses

Pancreatic masses can be broadly categorized into several types, including pancreatic adenocarcinoma, cystic neoplasms, and NETs. The most prevalent form, pancreatic adenocarcinoma, accounts for about 85-90% of all pancreatic malignancies and is characterized by an aggressive clinical course and poor prognosis [15]. Pancreatic cystic neoplasms, such as IPMNs, mucinous cystic neoplasms, and serous cystadenomas, represent another category with distinct epidemiological and pathological features. IPMNs and MCNs have malignant potential, and their identification often prompts further diagnostic and therapeutic intervention [16]. Lastly, NETs are rare but clinically significant neoplasms that vary widely in their biological behavior, from benign to highly malignant, and may secrete hormones causing unique clinical syndromes [9]. Each of these tumor types requires distinct diagnostic and therapeutic approaches, underscoring the importance of accurate classification for patient management.

Diagnostic evaluation of pancreatic masses

The diagnostic evaluation of pancreatic masses is a multifaceted process, essential for determining the nature and extent of the disease, guiding therapeutic decision-making, and assessing prognosis. This evaluation typically involves a combination of clinical assessment, advanced imaging modalities, tissue sampling, and staging criteria to establish a comprehensive picture of the patient’s condition. The intricate nature of pancreatic masses, combined with their often aggressive course, highlights the importance of thorough diagnostic protocols.

Clinical Presentation

Patients with pancreatic masses often present with a variety of nonspecific symptoms that can complicate early detection. The most common presentations include unexplained weight loss, jaundice, and abdominal pain. Weight loss is frequently observed in patients with PDAC and can result from tumor-associated cachexia, malabsorption, or metabolic disturbances [17]. Jaundice, particularly painless jaundice, is a classic sign of pancreatic head masses that obstruct the common bile duct, leading to elevated bilirubin levels [18]. Abdominal pain, often described as a dull ache that radiates to the back, is also prevalent and may indicate tumor infiltration into surrounding neural structures or organs. Other symptoms, such as nausea, fatigue, and early satiety, are common but nonspecific, often resulting in a delayed diagnosis due to their vague nature.

Imaging Studies

**Figure 1:**
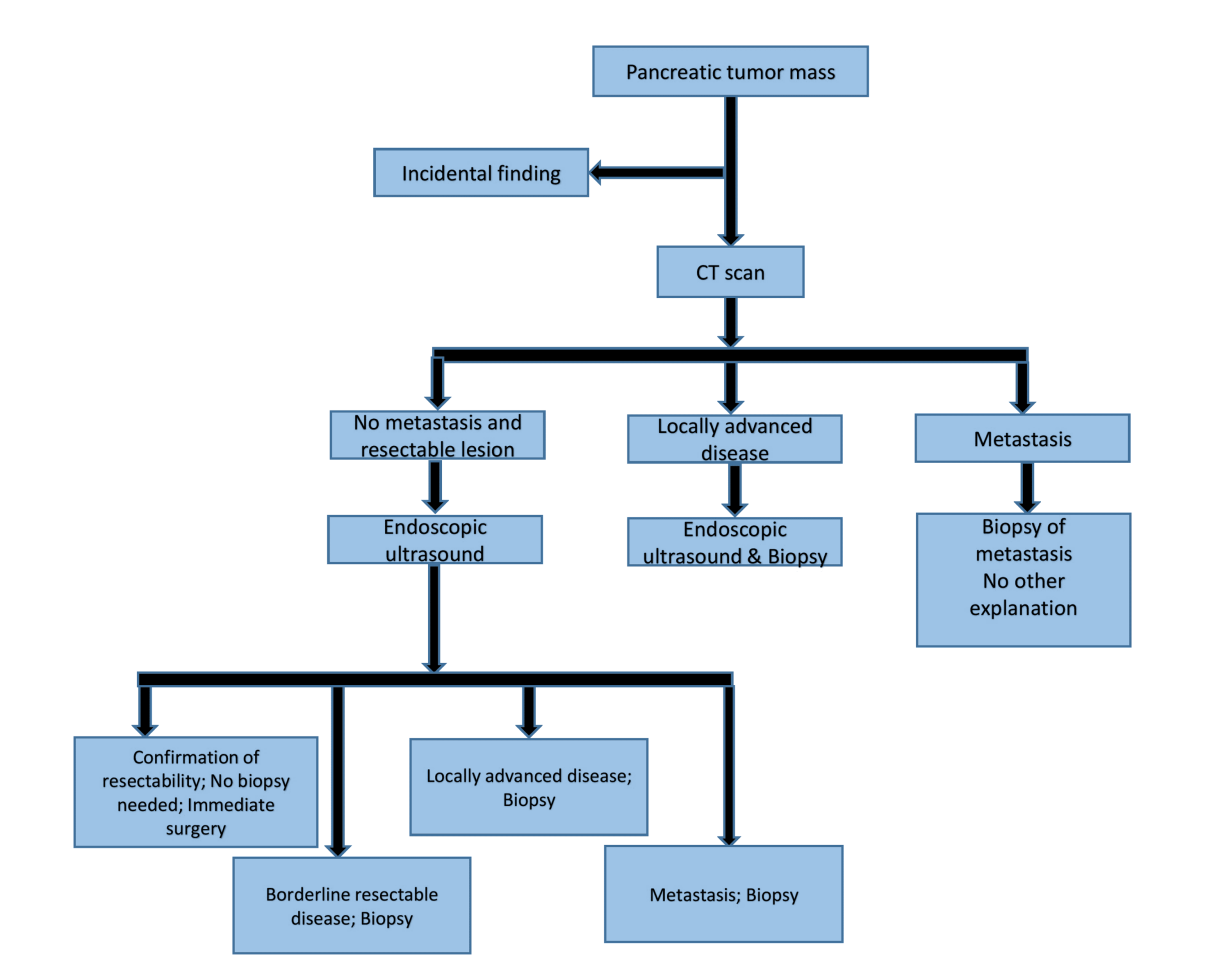
Diagnostic workup before the multidisciplinary decision. Figure created by Patricio Xavier Duran S.

Imaging plays a crucial role in the detection, characterization, and staging of pancreatic masses (Figure 1). Contrast-enhanced CT is typically the first-line imaging modality due to its high sensitivity in identifying pancreatic tumors and defining local extension, vascular involvement, and distant metastasis. CT scans allow for a detailed assessment of mass morphology, arterial and venous encasement, and lymph node involvement, which are critical for determining surgical resectability [19]. MRI and magnetic resonance cholangiopancreatography are additional tools, particularly useful for evaluating cystic lesions and providing high-resolution images of the pancreaticobiliary ductal system. MRI is advantageous in distinguishing between benign and malignant cystic lesions, which helps in preoperative planning and management [20].

EUS is another essential imaging modality, offering both diagnostic and therapeutic capabilities. EUS provides high-resolution images of the pancreas and allows for FNA of the mass for cytologic examination. This is particularly beneficial for evaluating small lesions that may not be clearly defined on CT or MRI. Additionally, EUS-FNA is valuable for obtaining tissue samples for histopathological analysis, aiding in tumor classification and guiding subsequent treatment strategies [21].

Biopsy and Histopathology

Histopathological evaluation through biopsy is integral for confirming the diagnosis of pancreatic masses and distinguishing between malignant and benign conditions. EUS-guided FNA biopsy is the preferred method for tissue sampling, given its minimally invasive nature and ability to target specific areas within the pancreas under direct visualization. The tissue samples obtained through FNA are then subjected to cytologic and immunohistochemical analyses, which allow for the classification of the tumor type, such as adenocarcinoma, NET, or cystic neoplasm [22]. In some cases, percutaneous biopsy may be utilized, particularly for large masses or those with peritoneal involvement; however, it is generally less favored due to potential complications such as tumor seeding along the needle tract.

Tumor Staging

The TNM staging system, established by the American Joint Committee on Cancer (AJCC), is the standard framework for classifying the extent of pancreatic tumors, guiding surgical planning and prognosis estimation [2]. The “T” component refers to the tumor’s size and extent of invasion into surrounding tissues, while the “N” component addresses lymph node involvement. The “M” component denotes distant metastasis, commonly to the liver or peritoneum in pancreatic cancer. Accurate staging is crucial for determining resectability, as only localized (Stage I-II) tumors are generally considered for surgical resection, whereas advanced stages may be more suitable for palliative therapies [2]. Preoperative staging through imaging and biopsy enables a tailored approach to each case, ensuring optimal therapeutic outcomes.

Surgical management

Resectable Neoplasms

Certain pancreatic neoplasms, both cystic and solid, may be amenable to surgical resection. Treatment decisions are guided by the histological diagnosis and the presence of symptoms. For asymptomatic benign neoplasms, treatment may not be necessary, and annual follow-up monitoring can be sufficient. However, surgical intervention is indicated for benign neoplasms that present with significant size, symptomatic progression, or potential for dysplasia, particularly when non-invasive treatments have failed to manage the symptoms effectively [23].

One example of a benign pancreatic neoplasm that may require surgical resection is IPMN. IPMNs carry the risk of malignancy through a series of genetic and morphological changes, with an estimated 19-30% risk of progression to cancer [24]. Prophylactic surgical resection in such cases has demonstrated substantial benefits, including an increase in five-year survival rates to as high as 77-100% [25].

Another significant type of precursor lesion is pancreatic intraepithelial neoplasia (PanIN), which can evolve into invasive PDAC, and thus, it is recommended to surgically resect the neoplasm to prevent malignant transformation [26,27]. Surgical resection of PanIN is recommended to mitigate the risk of malignant transformation.

For surgical eligibility, the neoplasm must be confined to the pancreas without invasion of adjacent blood vessels, limiting surgical candidacy to Stage I and II malignancies. Surgery remains the only potentially curative option for pancreatic cancer, yet only a small proportion of patients (15-20%) meet the criteria for resection at diagnosis. According to Dr. Puckett, despite successful surgical intervention, the prognosis remains poor, with up to 90% of patients succumbing to the disease within one year post-surgery [28].

Stage III locally advanced pancreatic tumors account for approximately 30% of cases and are generally considered unresectable due to metastatic spread or extensive involvement of critical vascular structures such as the superior mesenteric vein or portal vein. However, some tumors may be categorized as borderline resectable if there is potential for achieving R0 resection margins through comprehensive en bloc vascular resection (Table 1).

**Table 1:** International consensus of classification of borderline resectable pancreatic tumors.

Vascular/Anatomical structure	Resectable (R)	Borderline resectable (BR)	Unresectable (UR)	Metastatic (M)
Superior mesenteric vein/Portal vein	No contact or unilateral narrowing	Tumor contact ≥180° or bilateral narrowing/occlusion, not exceeding the inferior border of the duodenum (BR-PV)	Bilateral narrowing/occlusion exceeding the inferior border of the duodenum	Not applicable
Superior mesenteric artery/Celiac axis	No tumor contact	Tumor contact <180° without deformity/stenosis (BR-A)	Tumor contact ≥180° or arterial encasement	Not applicable
Common hepatic artery	No tumor contact	Tumor contact without involvement of the proper hepatic artery and/or celiac axis	Tumor contact with involvement of proper hepatic artery and/or celiac axis	Not applicable
Aorta	No tumor contact	No tumor contact	Tumor contact or invasion	Not applicable
Metastases	No distant metastases	No distant metastases	No distant metastases	Distant metastases present

Dr. Ferrone’s study underscored the efficacy of neoadjuvant therapy in conjunction with surgical resection for patients with stage III borderline resectable tumors. The study reported that 92% of patients initially deemed unresectable achieved R0 resection margins following successful combined treatment [29,30].

Pancreatectomy

Pancreatic resections are classified based on the portion of the pancreas removed [31]. Pancreatectomy refers to the excision of pancreatic tissue, which can be total or partial, depending on the extent of the neoplasm and the surgical approach required [31].

Partial pancreatectomy: Central pancreatectomy is indicated for neoplasms confined to the neck and body of the pancreas that have low malignant potential. This surgery is not recommended for neoplasms with high dysplastic potential or malignancy, as it may leave residual malignant tissue at the margins [31]. Additionally, lymph node resection is less thorough compared to more extensive surgeries, increasing the risk of residual cancer and recurrence [32].

However, for benign lesions, central pancreatectomy offers a clear advantage by preserving pancreatic function, thereby reducing the incidence of postoperative diabetes to 12%, compared to 50% in distal pancreatectomy patients. Only 10% of patients undergoing central pancreatectomy require enzyme supplementation post-surgery [32].

**Figure 2:**
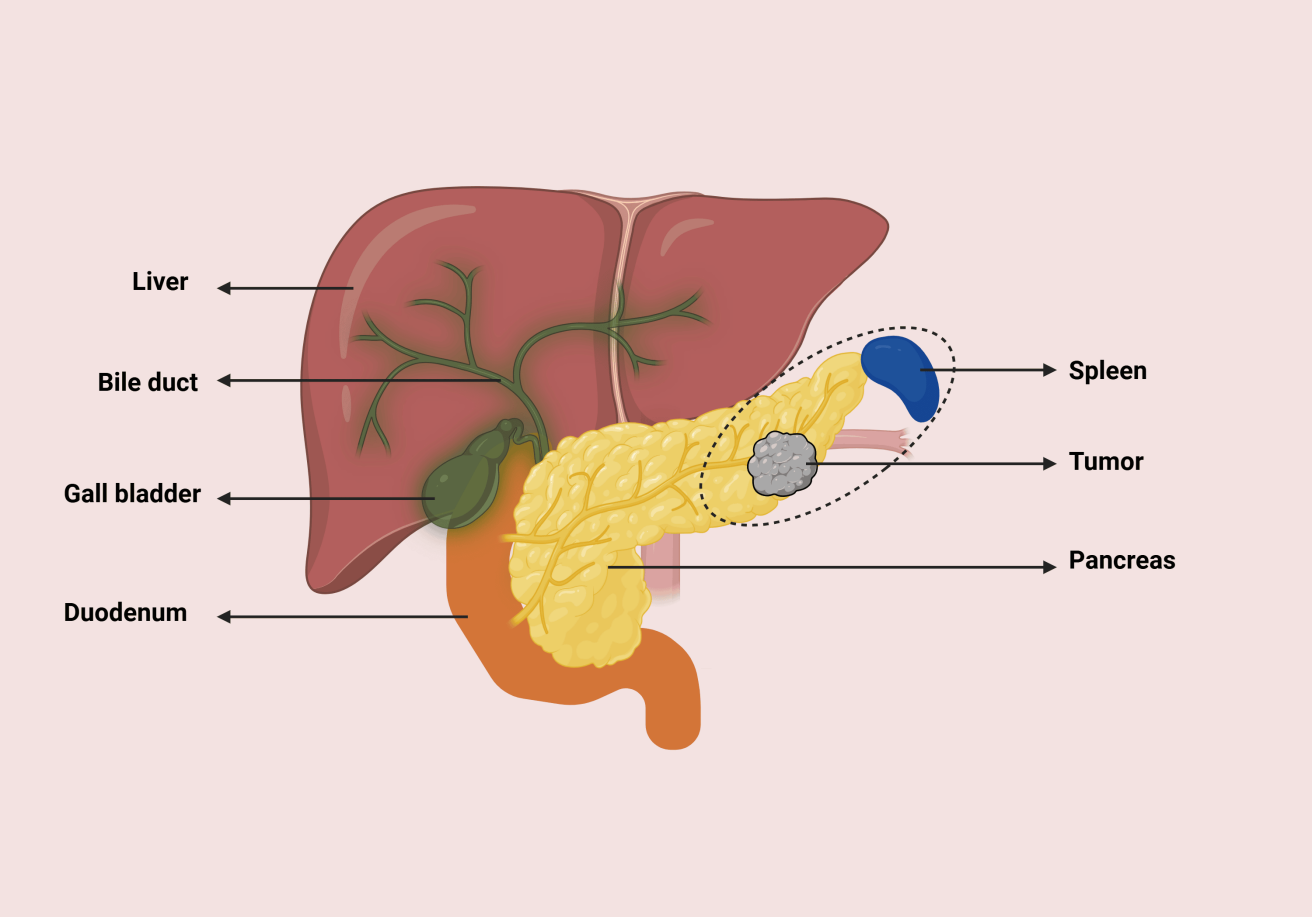
Distal pancreatectomy: removal of the body and tail of the pancreas and spleen. The dotted circle indicates the anatomical region (body and tail of the pancreas) to be removed during distal pancreatectomy along with the spleen. Figure created by Cara Mohammed.

Distal pancreatectomy is performed for neoplasms located in the body and tail of the pancreas (Figure 2). In cases of malignant neoplasms, nearby lymph nodes and the spleen are often resected as well. Splenectomy increases the risk of postoperative complications such as infections from encapsulated bacteria and sepsis. Patients undergoing distal pancreatectomy face a higher risk of pancreatic leakage at 30%, compared to 13% in pancreaticoduodenectomy [33].

All surgical interventions come with risks of complications such as fistulas, leaks, and hemorrhage, which can be minimized if performed by surgeons specializing in pancreatic surgery. Distal pancreatectomy has the advantage of preserving more pancreatic tissue compared to total pancreatectomy, leading to lower rates of postoperative supplementation, at around 27% [32]. A study by Ruess et al. indicated no significant difference in five-year survival rates between patients undergoing the Whipple procedure (17.8%) and those undergoing distal pancreatectomy (22%) [34].

**Figure 3:**
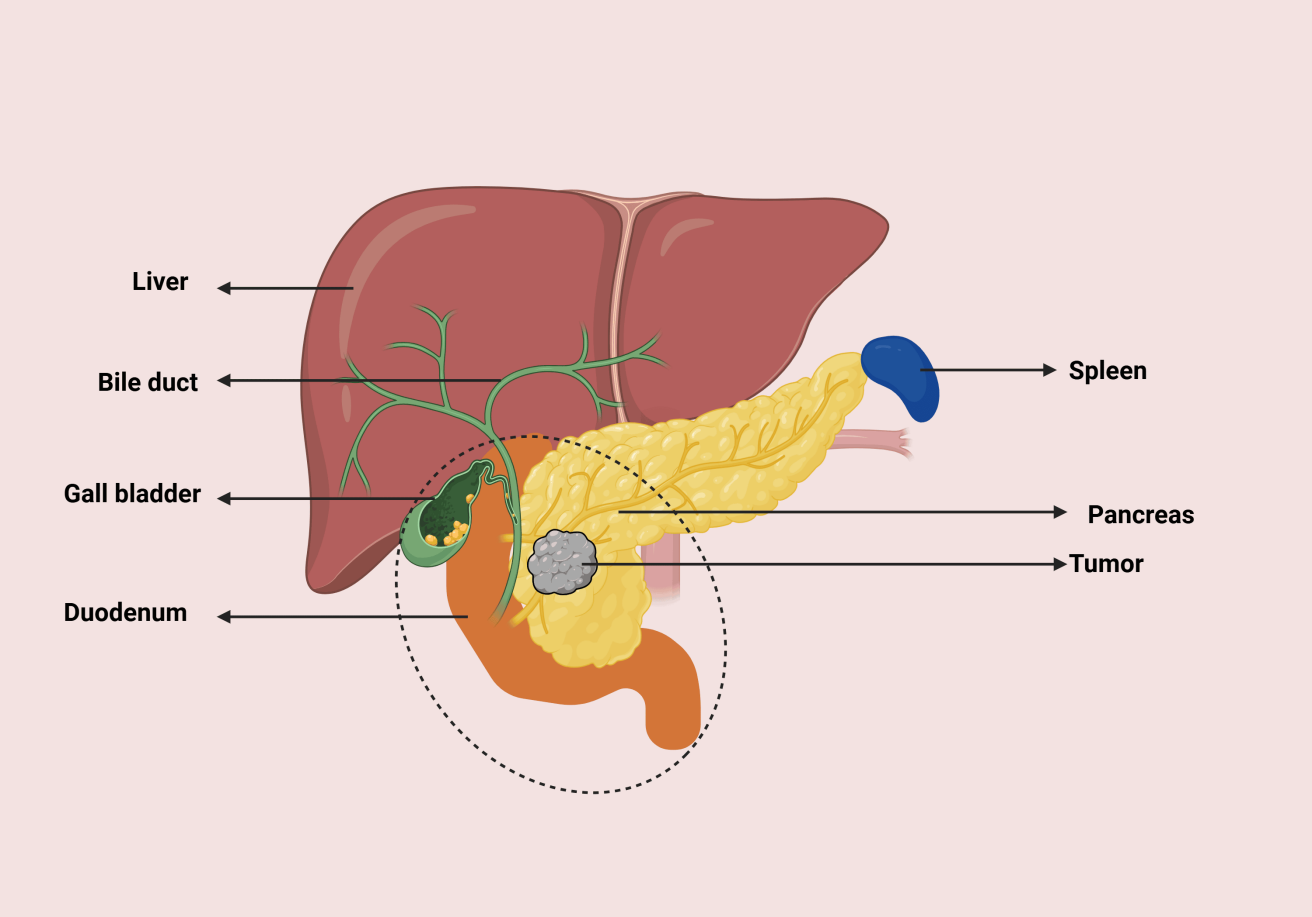
Whipple procedure: removal of the distal stomach, duodenum, and proximal jejunem; head of pancreas; gallbladder, and common bile duct. The dotted circle highlights the anatomical area removed during the Whipple procedure, including the head of the pancreas, duodenum, gallbladder, and part of the bile duct. Figure created by Hugh Kolomar.

Whipple procedure: Also known as a pancreatoduodenectomy, the Whipple procedure is a complex surgery performed to remove neoplasms confined to the head of the pancreas (Figure 3). The procedure involves the removal of the pancreatic head, gallbladder, duodenum, nearby lymph nodes, the distal bile duct, and sometimes part of the stomach, depending on the extent of the tumor. The procedure concludes with anastomoses to reconnect the remaining pancreas and bile duct to the small intestine, preserving digestive function.

The Whipple procedure can be performed using different surgical techniques, with the open surgical approach being the most common. This surgery has a one-year survival rate of 58.4% and a three-year survival rate of 34.8% [33]. Despite its potential benefits, it carries significant risk, with postoperative complications occurring in up to 51% of cases [35]. Approximately 5-15% of patients may die from surgical complications, and even those who recover can face postoperative infections, adhesions, bleeding, digestive issues, and diabetes. Lifelong insulin and pancreatic enzyme supplementation are often required, affecting quality of life.

A variation called pylorus-preserving pancreaticoduodenectomy removes only part of the duodenum and preserves the stomach’s pylorus [35]. This approach may be more suitable for severely malnourished patients, as it allows for more normal weight gain during recovery compared to the standard Whipple procedure, which can lead to greater weight loss [35].

Total pancreatectomy: A total pancreatectomy involves the complete removal of the pancreas, gallbladder, common bile duct, part of the stomach and small intestine, and the spleen, followed by the creation of anastomoses to maintain digestive function [33]. This surgery is primarily used for the complete removal of pancreatic cancer that has spread throughout the pancreas [33].

Although patients can survive without a pancreas by taking pancreatic enzyme replacements, they will develop diabetes that is challenging to manage due to total dependence on exogenous insulin. This condition significantly impacts the patient’s quality of life. Additionally, the removal of the spleen necessitates vaccination against encapsulated bacteria due to an increased risk of infections.

Currently, total pancreatectomy is not commonly performed because of its radical nature and the high rate of complications, coupled with the lack of evident survival advantages over less radical approaches [36]. Importantly, the removal of the pancreas does not guarantee a cure for cancer, as studies show postoperative survival rates of 41.4% at one year and 21.7% at three years. However, these rates can vary based on individual patient health [33].

Minimally Invasive Surgical Techniques

Minimally invasive surgery (MIS) of the pancreas was thought to be unsuitable for a long time. This perspective was challenged in 1994 by Michel Gagner, who performed the first laparoscopic pancreatic head resection [37]. The choice between laparoscopic, robotic, or open approaches depends on tumor location, size, vascular involvement, surgeon experience, and institutional resources. While laparoscopic surgery is generally preferred for distal pancreatectomy in benign or low-grade lesions, robotic-assisted surgery offers enhanced precision for complex resections, particularly in high-volume centers, whereas open surgery remains the standard for extensive vascular involvement or technically challenging tumors [38]. Numerous studies have demonstrated beneficial effects of MIS in terms of clinical outcomes such as reduced blood loss and shorter hospital stay over the open technique in the case of distal pancreatic resection [38,39]. These findings were confirmed in the LEOPARD trial as well. This study included 108 patients from 14 centers, and the results showed that compared with patients who underwent open distal pancreatectomy, patients who underwent minimally invasive distal pancreatectomy had better postoperative functional recovery and less delayed gastric emptying (DGE) [40].

Laparoscopic surgery: For the last two decades, laparoscopic pancreatic surgery has been significantly more popular. Studies consistently show that laparoscopic pancreatic surgery is safe and effective compared to open surgery [41,42]. A systematic review found various advantages associated with laparoscopic procedures compared to the open approach, such as lower complication rates (RR = 0.78), less blood loss (average 298 mL less), fewer days spent within the hospital (mean decrease by approximately three days), and a greater number of lymph nodes harvested. Although the operation duration for laparoscopic pancreatic surgery was slightly higher, this difference was not clinically significant [43]. When comparing laparoscopic distal pancreatectomy with open distal pancreatectomy, laparoscopic distal pancreatectomy often results in a shorter hospital stay and a lower chance of DGE [44,45]. However, laparoscopic distal pancreatectomy can extend operating time and may lead to higher incidences of postoperative pancreatic fistula (POPF) and readmission [46,47].

Robotic-assisted surgery: Robotic surgery offers greater dexterity and range of motion [48]. Since the da Vinci robot received approval in 2000, there has been renewed interest in using this alternative platform for pancreaticoduodenectomy [49]. Advances such as three-dimensional imaging, instruments with near 360-degree articulation, and improved precision have spurred a significant increase in robotic-assisted surgeries [50-52]. Studies show that while the robotic approach requires a longer operative time, averaging 491.5 minutes compared to 264.9 minutes for open procedures, it also results in less blood loss (mean of 247 mL vs. 774.8 mL) and a shorter hospital stay (mean of 13.7 days vs. 25.8 days) compared to open surgery [53]. In a comprehensive North American study across multiple high-volume centers, robotic pancreaticoduodenectomy showed longer operative times but a reduction in major complications compared to open distal pancreatectomy, even when performed by experienced surgeons [54].

Conversion from minimally invasive pancreaticoduodenectomy to open surgery has been linked to adverse outcomes. Some studies indicate that conversion rates are generally lower with robotic surgery compared to traditional laparoscopy [55,56]. Robotic partial hepatectomy also shows promisingly low conversion rates. For instance, a study by Tsung et al. compared robotic (n = 57) and laparoscopic (n = 114) hepatectomies. Their findings showed that the robotic approach achieved a completion rate of 93% compared to 49% with laparoscopy (p < 0.001), with similar results across both major (81% vs. 7%, p < 0.001) and minor hepatectomies (100% vs 75%, p = 0.013) [57]. Although reports affirm the safety and feasibility of both robotic and laparoscopic techniques, there is still debate about their respective roles and benefits in hepato-pancreato-biliary surgery [58].

Palliative surgical procedures: For patients with advanced pancreatic cancer or tumors that are not resectable, palliative surgical procedures play a crucial role in managing symptoms, improving quality of life, and maintaining gastrointestinal function. These interventions do not aim to remove the tumor entirely but rather to alleviate symptoms such as obstructive jaundice, digestive issues, and pain. Palliative surgery is especially important for patients with tumors compressing or invading adjacent organs, where tumor removal is not feasible due to the extent of spread [59]. A systematic review on palliative surgery to remove the primary tumor in cases of unresectable metastatic small intestinal and pancreatic NETs found that patients who underwent resection lived significantly longer [60].

Biliary bypass procedures: Biliary bypass is recommended in most instances for pancreatic cancer patients with obstructive jaundice [61-62]. In a biliary bypass, surgeons create a new pathway for bile flow from the liver to the intestine, bypassing the blocked bile duct and alleviating jaundice symptoms. The two most common forms of biliary bypass are choledochojejunostomy and cholecystojejunostomy [63]. The choice of procedure depends on the tumor’s location and the extent of ductal involvement. Biliary bypass procedures effectively relieve jaundice and improve the patient’s comfort and ability to digest food. A study involving 126 patients with pancreatic head carcinoma reported that Roux-en-Y hepaticojejunostomy combined with gastrojejunostomy provides effective relief for unresectable pancreatic head cancer. Furthermore, this approach has low mortality rates and manageable complications [64]. Compared to palliative stenting, biliary bypass surgery tends to provide longer-lasting relief from jaundice symptoms [65]. However, Glazer et al., in their systematic review, did not observe any significant difference in patient outcomes in bypass and stent placement groups in malignant biliary obstruction [66]. While generally safe, biliary bypass surgeries are still significant procedures and carry potential risks, including infection and bile leaks [67].

Palliative stenting for non-resectable tumors: The use of palliative endoscopic stents for gastric outlet obstruction began in the 1990s [68]. The process involves passing a guide wire through the narrowing or blockage, and with the help of fluoroscopic imaging, a stent is positioned to cover the obstruction [68]. There are various types of stents available, including self-expanding metal stents, which can be either covered or uncovered. Covered stents tend to lead to fewer instances of obstruction and, consequently, require less frequent reintervention [68]. Stent design is advancing, with partially covered stents now under investigation. Endoscopic palliation offers a well-tolerated and minimally invasive alternative to palliative surgery [69]. Fiori et al. observed that endoscopic stenting showed clear benefits, particularly with shorter operative times, faster return to eating, and shorter hospital stays [70]. Mehta et al. also found positive results, with patients showing improved physical health one month after duodenal stenting (p < 0.01) [71]. Two systematic reviews and meta-analyses found that both endoscopic stenting and palliative surgery achieve similar technical success and clinical outcomes. However, endoscopic stenting is associated with shorter hospital stays and faster recovery, making it preferable in patients with limited life expectancy or poor performance status. In contrast, surgical bypass offers superior long-term patency with significantly lower reintervention rates, making it more suitable for patients expected to survive longer than three to six months. Studies have shown that up to 20-30% of patients with stents may require reintervention due to stent occlusion or migration, whereas surgical bypass procedures maintain biliary or gastric patency more reliably over time [68]. Patients who received an endoscopic stent had a shorter hospital stay, while those who underwent surgery had a lower rate of needing further interventions [68,72]. Table 2 describes the existing techniques for pancreatic tumor resection.

**Table 2:** Different techniques for pancreatic tumor resection.

Technique	Advantages	Disadvantages	Key evidence
Minimally invasive surgery	Reduced blood loss; shorter hospital stay; faster recovery	Technically challenging for complex cases; requires specialized training; surgery of the pancreatic head is very challenging [73]	The LEOPARD trial [40] confirmed better recovery and less delayed gastric emptying with minimally invasive distal pancreatectomy compared to open distal pancreatectomy
Laparoscopic pancreatic surgery (LPS)	Reduced complication rates; less blood loss; fewer days in the hospital; more lymph nodes harvested	Slightly longer operation time; higher risk of postoperative pancreatic fistula [46,47]	Consistently shows safety and efficacy in multiple studies [43,45]. LPS reduces hospital stay and complications compared to open surgery
Robotic-assisted surgery	Greater dexterity; enhanced 3D imaging; less blood loss; shorter hospital stay	Longer operation time; expensive; requires trained personnel [74]	Robotic surgery reduces major complications and blood loss but requires significantly longer operative time than open surgery [53,54]
Palliative surgical procedures	Relieves symptoms (e.g., jaundice); improves quality of life	Significant surgical complication risk including cholangitis, delayed gastric emptying, and gastric outlet obstruction [75]	Palliative surgery in unresectable tumors improves life quality and symptoms but is associated with surgical risks [60]
Biliary bypass procedures	Long-lasting relief for obstructive jaundice; reduces the need for reintervention	Risk of infection and bile leakage; eequires more extended hospital stay than stenting [67]	Biliary bypass provides durable relief for jaundice; however, it may involve more complications [67]
Palliative stenting	Minimally invasive; shorter operative time and faster recovery	May require reintervention; potential stent migration or occlusion [76]	Stenting offers an effective, quick relief option with fewer complications and quicker recovery compared to bypass surgery [68,70-72]

Role of a multidisciplinary approach in surgical management

Effectively managing pancreatic masses necessitates a comprehensive, multidisciplinary approach that incorporates expertise from various medical specialties. The global rise in pancreatic cancer cases has underscored the importance of enhancing our understanding of its fundamental mechanisms while advancing treatment strategies. This includes exploring innovative and integrated approaches to treatment aimed at improving patient outcomes through personalized care plans.

Large medical centers currently leverage the expertise of multiple clinical departments, including pancreatic surgery, gastroenterology, oncology, radiotherapy, pathology, medical imaging, and nuclear medicine. Despite valuable inputs from each specialty, limitations remain, making a fully integrated multidisciplinary strategy essential for addressing all aspects of treatment, from decision-making and surgery to chemoradiotherapy and targeted therapies [77].

A study by Morales et al. at a tertiary care center highlighted the benefits of a multidisciplinary approach in the management of pancreatic cancer [78]. The integration of expertise from surgeons, gastroenterologists, radiologists, oncologists, and pathologists significantly enhanced tumor resectability assessments, achieving a concordance rate of 91% and a surgical resectability rate of 24.1%. Coordinated care also reduced the frequency of unnecessary exploratory surgeries and improved patient outcomes, resulting in a median survival of 24 months.

Research conducted by Quero et al. emphasized the role of multidisciplinary tumor boards (MDTBs) in optimizing pancreatic cancer management. Collaborative assessments facilitated by MDTBs led to improved treatment plans and a high concordance rate of 91.5% between MDTB evaluations and intraoperative findings, enhancing surgical decision-making and patient outcomes [79]. Although MDTBs have proven beneficial, further research is warranted to fully understand their long-term impact.

Postoperative management and complications

The postoperative management of pancreatic neoplasms is complex, requiring an interdisciplinary approach to address immediate recovery, complications, and long-term outcomes. Advancements in surgical techniques, perioperative care, and adjuvant therapies have significantly improved patient outcomes, although challenges such as POPF, infections, and recurrence persist. Personalized care plans tailored to individual risk factors and complications are essential for optimizing recovery and enhancing the quality of life for these patients.

Immediate Postoperative Care

The immediate postoperative phase is critical for ensuring recovery, minimizing complications, and facilitating a return to normal functions. Patients are closely monitored in the intensive care unit immediately following surgery to assess vital signs, manage pain, and detect early complications. Early mobilization is encouraged to prevent deep vein thrombosis and respiratory infections [80]. Pain management strategies, including epidural analgesia or patient-controlled analgesia, play a pivotal role in enhancing recovery [81].

Nutritional support is an integral part of postoperative care. Many patients require enteral or parenteral nutrition initially, transitioning to oral intake as tolerated. A jejunal feeding tube is often placed intraoperatively to provide nutritional support while bypassing the anastomosis [82]. Enteral feeding is preferred over parenteral options due to its role in maintaining gut integrity and reducing infection risks. Nutritional assessments and interventions by dietitians are essential to address malabsorption and weight loss commonly associated with pancreatic surgeries [83].

Effective wound care reduces the risk of surgical site infections. Daily wound assessments are performed to monitor for erythema, discharge, or dehiscence [84]. Antibiotics may be administered prophylactically or in response to signs of infection. Open drainage of intra-abdominal collections may also be required in cases of significant contamination or abscess formation [84].

Common Postoperative Complications

Despite advancements in surgical techniques, complications following surgery for pancreatic neoplasms remain prevalent and challenging.

POPF, defined as the leakage of pancreatic fluid from the anastomosis site, is the most significant complication. It occurs in approximately 15-30% of patients and contributes to sepsis, intra-abdominal abscesses, and prolonged hospital stays [85]. The most notable risk factor for POPF is a soft pancreatic texture, which is associated with increased enzymatic leakage due to fragile tissue at the anastomotic site. Other contributing factors include small pancreatic duct size (<3 mm), high body mass index, excessive intraoperative blood loss, and the type of surgical reconstruction. Risk assessment tools such as the Fistula Risk Score and its modified versions incorporate these variables, including gland texture, duct size, blood loss, and pathology, to predict the likelihood of clinically relevant POPF [85]. Early detection involves monitoring amylase levels in abdominal drains [86]. Management includes nutritional support, antibiotics, and, in severe cases, interventional radiology or reoperation.

DGE, characterized by the inability to tolerate oral intake due to gastric dysmotility, occurs in about 10-30% of cases [87]. Contributory factors include postoperative inflammation, pyloric dysfunction, intra-abdominal infections, and extensive lymphadenectomy. Risk is notably increased in patients with POPF or other complications that delay postoperative gastrointestinal recovery [88]. Prokinetic agents, such as metoclopramide, and dietary modifications often aid recovery. Assessment of risk is largely clinical, but recent research supports predictive modeling using preoperative factors and postoperative biomarkers [87,88]. Persistent DGE may necessitate endoscopic interventions [88].

Surgical site infections, intra-abdominal abscesses, and pneumonia are common postoperative infections [89]. Risk factors include prolonged operative times, malnutrition, and immunosuppression. Early diagnosis and targeted antibiotic therapy are critical. In severe cases, percutaneous drainage of abscesses may be required [88].

Long-Term Outcomes

Surgical resection of pancreatic neoplasms often results in significant physiological changes, including endocrine and exocrine insufficiency [90]. Patients may experience malnutrition, weight loss, and fatigue. Endocrine insufficiency can lead to insulin-dependent diabetes mellitus, requiring intensive glycemic management. Pancreatic enzyme replacement therapy is essential for managing exocrine insufficiency and improving nutrient absorption [91]. Psychological support, including counseling and support groups, is crucial for addressing the emotional impact of a cancer diagnosis and its treatment [92].

The recurrence of pancreatic neoplasms remains a significant challenge, with recurrence rates as high as 50-80% within two years of surgery [93]. Recurrence may be local, at the surgical site, or distant, involving the liver or peritoneum. Regular follow-ups with imaging studies, tumor marker assessments (e.g., CA 19-9), and clinical evaluations are vital for early detection. Adjuvant chemotherapy, often with agents such as gemcitabine or FOLFIRINOX, is recommended for patients at high risk of recurrence to improve survival outcomes [94].

Adjuvant and neoadjuvant therapies in conjunction with surgery

Surgical resection remains the definitive curative treatment for pancreatic neoplasms, with chemotherapy and radiotherapy serving as crucial adjunctive modalities. Treatment strategies are primarily determined by assessing whether the disease is “potentially curable,” which is based on the extent of vascular invasion rather than metastatic status.

The surgical curability of the neoplasm is determined by the extent of invasion of local vasculature, regardless of signs of metastases. Resectable tumors approximate the AJCC’s (American Joint Committee for Cancer’s TNM classification) staging scheme. Resectable tumors, typically AJCC Stage I or II, are surgically removed and followed by adjuvant chemotherapy to eliminate residual malignant cells and prevent recurrence [95]. “Borderline resectable” tumors, usually AJCC Stage II or III, present with vascular involvement that complicates achieving disease-free margins [96]. These cases typically undergo neoadjuvant chemotherapy before surgery to reduce tumor size and improve resection feasibility [97].

The role of neoadjuvant therapy is evolving in both resectable and borderline resectable diseases. Its theoretical advantages include better control of micrometastases and delivery of potent chemotherapy before surgical stress [98]. Additionally, it serves as a diagnostic tool, with tumor response indicating disease aggressiveness [99]. This approach can potentially convert previously unresectable tumors to resectable status while preventing unnecessary surgeries in non-responsive cases. Post-surgery radiation therapy is considered for patients with positive margins or residual disease after adjuvant chemotherapy [100].

Studies have demonstrated that neoadjuvant therapy enhances overall survival and surgical outcomes for patients with borderline resectable pancreatic cancer [101]. Nevertheless, more research is needed to determine its impact on patients with resectable PDAC [79,101]. Secondary outcomes, such as disease-free survival, R0 resection rates, and lower incidences of pathological lymph node involvement, perineural invasion, and venous invasion, have all shown improvement following neoadjuvant therapy [102,103].

However, the PREOPANC trial, which relied on single-agent gemcitabine chemotherapy, highlighted the limited long-term survival benefits, emphasizing the need for more advanced treatment protocols [104]. Additionally, while immune checkpoint inhibitors have shown promise in treating certain solid tumors, their effectiveness in pancreatic cancer has been limited. A recent phase II randomized trial reported suboptimal outcomes using the combination of durvalumab (an anti-programmed death-ligand 1 agent) and tremelimumab (an anti-cytotoxic T-lymphocyte-associated protein 4 agent) for metastatic pancreatic cancer [105].

Immunotherapy and targeted therapy currently play limited roles in treatment. FDA approval exists for pembrolizumab in patients with *BRCA* 1/2 mutations or MMR deficiencies, while olaparib is under review as maintenance therapy for *BRCA* 1/2 mutation cases [106]. Future treatment directions focus on CAR-T and ATCT strategies, particularly targeting *KRAS* mutations, which show promising preclinical results [107].

The CONKO-001 study demonstrated the significant benefits of combined treatment approaches [108]. In this study, patients receiving adjuvant chemotherapy showed nearly doubled five-year survival rates (20.7% vs. 10.4%) and improved ten-year survival by 4.5% compared to surgery alone. Disease-free survival time also doubled from 6.7 to 13.4 months. These results established combination therapy as standard practice and prompted further research into optimizing chemotherapy regimens.

Advances in surgical techniques and future directions

Robotic surgery has emerged as a transformative approach in the surgical management of pancreatic neoplasms [109]. Robotic systems offer enhanced dexterity, high-definition 3D visualization, and tremor filtration, enabling precise dissection and reconstruction. These features are particularly advantageous in minimally invasive pancreaticoduodenectomy and distal pancreatectomy, reducing operative times, blood loss, and recovery durations. Studies have also highlighted comparable oncological outcomes to open surgery, making robotic platforms increasingly preferred [110].

Intraoperative imaging has further revolutionized pancreatic surgery by providing real-time anatomical and functional insights. Techniques such as indocyanine green fluorescence imaging are now commonly employed to assess tissue perfusion, identify critical structures, and ensure complete tumor resection [111]. Additionally, intraoperative ultrasound aids in localizing small lesions and defining tumor margins, contributing to better surgical accuracy [112].

Emerging technologies are paving the way for safer and more effective surgical interventions. Augmented reality (AR) and 3D printing are being explored to create patient-specific models for preoperative planning, allowing surgeons to visualize complex anatomical relationships before and during surgery. Laser-based systems and advanced energy devices have also enhanced the precision of pancreatic resections, reducing collateral damage to surrounding tissues [113]. However, widespread clinical adoption of these innovations faces several barriers, including high implementation costs, limited accessibility in low-resource settings, and the need for specialized training. Moreover, many of these technologies, including artificial intelligence (AI)-driven platforms and AR-assisted surgery, are still undergoing clinical validation, with insufficient large-scale data to establish standardized protocols.

Additionally, the integration of AI in surgical decision-making is showing promise. AI-driven tools analyze large datasets to predict surgical risks, suggest optimal strategies, and anticipate complications, contributing to personalized and adaptive intraoperative workflows [114].

The era of precision medicine has brought a paradigm shift in the surgical management of pancreatic neoplasms. Genetic and molecular profiling now guide tailored approaches to tumor resection. For instance, *BRCA*-mutated pancreatic cancers may benefit from neoadjuvant therapies such as PARP inhibitors before surgery to enhance resectability [115]. Similarly, molecular markers such as *KRAS* mutations or DNA mismatch repair status help identify patients likely to respond better to specific adjuvant therapies, optimizing outcomes post-surgery [116].

## Conclusions

Effective management of pancreatic masses hinges on early diagnosis, accurate classification, and timely surgical intervention. While resection remains the only curative option, advances in minimally invasive and robotic-assisted techniques are improving outcomes. Neoadjuvant therapy and multidisciplinary care are enhancing resectability and survival, while emerging technologies such as AI and precision medicine are paving the way for more personalized treatment strategies. To improve clinical outcomes, robotic-assisted surgery should be prioritized in high-volume centers where adequate expertise and infrastructure are available, especially for complex resections requiring precision. Institutions should establish formal MDTBs to assess resectability, personalize treatment strategies, and minimize unnecessary exploratory surgeries. Surveillance protocols incorporating periodic imaging and serum CA 19-9 monitoring should be standardized postoperatively to enable early detection of recurrence. Furthermore, efforts must focus on validating emerging technologies and expanding their accessibility across diverse healthcare settings to ensure equitable implementation.
